# Impedance-based forecasting of lithium-ion battery performance amid uneven usage

**DOI:** 10.1038/s41467-022-32422-w

**Published:** 2022-08-16

**Authors:** Penelope K. Jones, Ulrich Stimming, Alpha A. Lee

**Affiliations:** 1 Department of Physics, University of Cambridge, Cambridge, UK; 2 The Alan Turing Institute, London, UK; 3 Chemistry, School of Natural and Environmental Sciences, Newcastle University, Newcastle upon Tyne, UK

**Keywords:** Batteries, Cheminformatics, Applied mathematics, Scientific data, Energy storage

## Abstract

Accurate forecasting of lithium-ion battery performance is essential for easing consumer concerns about the safety and reliability of electric vehicles. Most research on battery health prognostics focuses on the research and development setting where cells are subjected to the same usage patterns. However, in practical operation, there is great variability in use across cells and cycles, thus making forecasting challenging. To address this challenge, here we propose a combination of electrochemical impedance spectroscopy measurements with probabilistic machine learning methods. Making use of a dataset of 88 commercial lithium-ion coin cells generated via multistage charging and discharging (with currents randomly changed between cycles), we show that future discharge capacities can be predicted with calibrated uncertainties, given the future cycling protocol and a single electrochemical impedance spectroscopy measurement made immediately before charging, and without any knowledge of usage history. The results are robust to cell manufacturer, the distribution of cycling protocols, and temperature. The research outcome also suggests that battery health is better quantified by a multidimensional vector rather than a scalar state of health.

## Abstract (web summary)

Accurate forecasts of lithium-ion battery performance will ease concerns about the reliability of electric vehicles. Here, the authors leverage electrochemical impedance spectroscopy and machine learning to show that future capacity can be predicted amid uneven use, with no historical data requirement.

## Introduction

Electrification of the transportation industry is now taking place at an increasingly rapid pace, enabling significant strides towards a carbon neutral future. Fundamental to this transition has been the development of the lithium-ion battery, which powers the majority of electric vehicles (EVs) on the road today. Notwithstanding the environmental benefits of this transition, reliance on the lithium-ion battery poses significant challenges, with consumer concerns including range anxiety, fear of battery failure and charging time. Easing these concerns demands the ability to accurately forecast battery performance, and specifically when usage conditions are variable.

The key challenge is the heterogeneity of the battery. Each user uses their car differently, and even across a single battery pack not all cells are necessarily charged or discharged with identical current^1–3^. These differences mean that each cell’s internal state, including the extent of lithium plating or electrode cracking, can vary significantly both at an intra-pack and inter-pack level^4,5^.

To quantify the extent of degradation within cells, and to identify cells that have reached their End of Life (in EVs, this is typically defined as the point at which the discharge capacity has reduced to 80% of the nominal capacity^6,7^), the scalar State of Health (SOH) metric is typically adopted, measured using previous cycle discharge capacity or internal resistance^8–13^. The problem with this approach is that batteries with the same numerical SOH do not necessarily exhibit identical levels of each degradation process (for example, lithium plating or electrode cracking), yet the impact of future cell usage on the cell’s future performance and degradation pathway depends significantly on the type of degradation that has already occurred^14–16^. Accurate forecasting of battery performance demands a non-invasive approach to acquire information about the cell state at a microscopic level.

Both short^11,17–20^ and long^21–23^ timescale forecasting of battery performance are of interest in battery prognostics. Over a short timescale, predicting how the battery would respond to a particular charging and discharging protocol can be used to develop optimal charging protocols^17^. Short-term forecasting also encompasses SOH estimation^11,18–20^: here, the aim is to predict the battery’s discharge capacity or internal resistance under a specific, standardised cycling protocol. Over a long timescale, the focus is on predicting the remaining useful life^21^, end of life^22^, or the ‘knee-point’ in the battery’s life trajectory at which degradation accelerates^23^.

Approaches to both types of forecasting can be subdivided into empirical, physics-based, and data-driven models, with some models being a hybrid of these. Empirical approaches have been used to model long-term capacity fade with power laws but assume fixed operation over battery life and do not account for intrinsic differences in cell state at start of life. These approaches assume that all cells of the same chemistry will fade in the same way if operated in the same way, which is not observed in practice^24^. In physics-based approaches, the battery is either modelled mechanistically using first principles analysis of internal physical and electrochemical processes, or using equivalent circuit modelling, which models the cell as a circuit comprising resistors and capacitors that are representative of the underlying electrochemical processes^25,26^. Mechanistic models aim to capture how the battery voltage responds to an externally applied current (or vice versa), which can be used to predict optimal charging protocols^17^. However, the parameters of such models need to be updated for each individual cell and typically suffer from non-identifiability – several sets of model parameters could explain the observed data equally well, but would make drastically different predictions on test cells or on the same cell later in its life. For circuit-based models, the parameters of the circuit can be fitted to either current-voltage data^20,27^, or to electrochemical impedance spectra^28,29^. The circuit parameters can then be used to forecast capacity degradation under standardised use conditions^20^ or to simulate the effect of different usage conditions on battery pack performance^30^. However, it is challenging to capture every degradation mode in an analytical model. Further, a new set of model parameters must be learnt for each cell from cycle to cycle, making it challenging to infer a general cell-to-cell model.

**Fig. 1:**
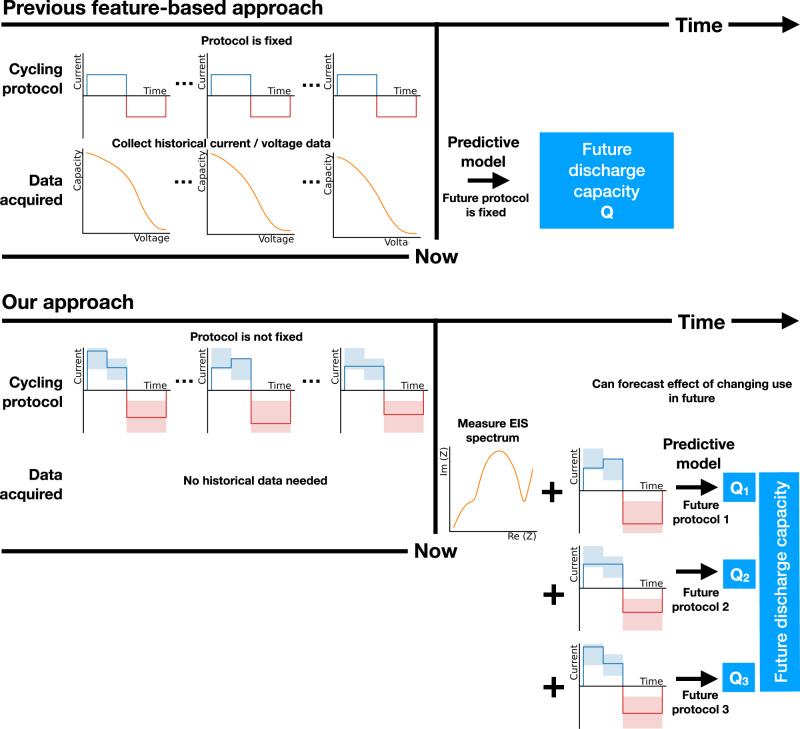
Schematic comparison of the proposed approach to previous research works. Feature-based methodologies for degradation prediction have focused on constant charging protocols (the blue/red curve denotes the charge/discharge phase), using features from capacity–voltage curves as input^22,51^. This necessitates knowledge of historic charging data. Our approach considers variable charging protocols (the shaded blue/red region denotes the range of currents that the charge/discharge protocols are drawn from), which is more comparable to the EV setting. Further, we employ the electrochemical impedance spectrum measured just before charging as input, without any knowledge of historic data, and predict the impact of different future usage protocols on the discharge capacity.

Purely data-driven approaches to forecasting use raw data as input to a machine learning algorithm to forecast long term capacity fade, resistance increase and remaining useful life^31,32^. Feature-based data-driven approaches applied machine learning on features extracted from the charging or discharging curve to predict discharge capacity^19^, remaining useful life^22^, and abrupt capacity decays^23,33^. Innovations in extracting features from charge/discharge curves^34^ and machine learning approaches for modelling time-series data^35,36^ have enabled significant improvements in the accuracy of predictions. Further studies showed that using features of the discharge curve across a small number of initial cycles, it is possible to train machine learning models that can generalise to different cell chemistries^37^. Going beyond charging and discharging curves, approaches such as electrochemical impedance spectroscopy (EIS)^21^, early cycle Coulombic efficiency^38^, current interruption^39^ and acoustic time-of-flight analysis^18,40^ have been used for degradation forecasting. These approaches provide a fuller description of battery state – for example, EIS captures the response of the cell over a broad frequency range, with different frequencies correlating to distinct physical, chemical and mechanical changes in the active material^26,41–43^. Data-driven methods typically utilise data generated in the laboratory setting, where cells are charged and discharged in the same way over the entirety of their lifetimes, thus the impact of variable cell usage on future performance can be ignored (see Fig. 1). However, extrapolating the models developed for laboratory setting to field data or other realistic usage profiles such as the Worldwide Harmonized Light Vehicles Test Cycles (WLTC)^13,44,45^, where cells are cycled in vastly different ways over their lifetimes, has proved a major challenge^7^.

In this work, we seek to identify whether there exists a sufficiently informative marker of cell health that can be used to forecast short-term and longer term future performance, amid uneven historical and future cell usage. Figure 1 provides an illustration of our approach, and how it differs from previous approaches. We find that upon acquisition of an EIS spectrum just before charging, both next cycle and longer term cell capacity can be predicted with a test error of less than 10%. When testing on cells subjected to similar cycling conditions to those used to train the model, our model achieves comparable accuracy to state-of-the-art forecasting models (8.2% test error versus 8.8% test error), except that our model enables forecasting with no access to any historical data, whereas previous state-of-the-art models require historical data from the cell’s cycling trajectory. In addition, when extrapolating to different operating temperatures, our model significantly outperforms the state-of-the-art model, achieving a 57% reduction in test error (from 34.2% to 14.6%).

We observe that our model is data-efficient, requiring just eight cells to attain a test error of less than 10%. Crucially, our approach is robust to dataset shift, attaining a test error of less than 7% on a dataset with a different distribution of cycling patterns to the training set. This is important for deployment in the field where driving patterns may be different from those used to train the model. We additionally demonstrate that, if available, using additional features based on historical capacity–voltage data can serve to augment the state representation and reduce average test error by up to 25%. Our approach is robust with respect to cell manufacturer, average usage pattern and operating temperature.

Further, our work fills a gap in publicly available data by contributing a large corpus of cycling data on cells under dynamic working conditions^46^. Our work focuses on a set of idealised usage distributions rather than realistic driving profile in order to demonstrate the extent of generalisability of the model. Our work departs from the NASA randomised usage dataset^47^, which randomly cycles cells for 50 cycles before measuring the next cycle discharge capacity after charging via a ‘reference’ protocol. Although several models for forecasting degradation under randomised conditions have been built based on this data^12,19,48^, the effect of a single protocol on next cycle discharge capacity cannot be disentangled, and there is a need for a reference charge/discharge protocol every few cycles which does not concord with typical field usage.

## Results

### Data generation

For this study, we generate two separate datasets corresponding to commercial LiR coin cells purchased from two different manufacturers, which allows us to test whether our approach is robust with respect to cell manufacturer.

**Fig. 2:**
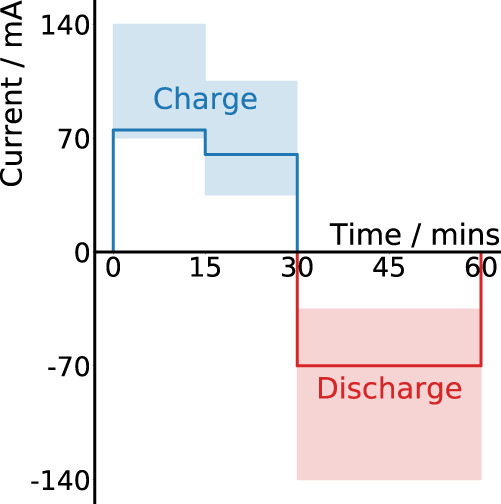
Proposed charge-discharge protocol. We generate battery cycling data by subjecting cells to a sequence of random charge and discharge currents. We apply two stages of constant current (CC) charging for up to 15 min each, with currents drawn from the ranges 70–140 mA (2–4 C) and 35–105 mA (1–3 C), respectively (the blue shaded region). If the safety threshold voltage of 4.3 V is reached before the time limit, then charging is stopped. During discharging, a single constant discharge current, randomly selected in the range 35–140 mA (1–4 C), is applied (the red shaded region), until the voltage drops to 3.0 V.

The first dataset corresponds to 40 Powerstream LiR 2032 coin cells (nominal capacity 1C = 35 mAh). We subject 24 cells to a sequence of randomly selected charge and discharge currents at 23 ± 2 °C for 110–120 full charge/discharge cycles. Each cycle consists of an initial diagnosis of battery state, involving acquisition of the galvanostatic EIS spectrum, followed by usage, involving a charging and discharging stage. Charging and discharging consist of a two stage and one stage Constant Current (CC) protocol, respectively; the currents are randomly selected at each cycle in the ranges 70–140 mA (2–4 C), 35–105 mA (1–3 C), and 35–140 mA (1–4 C) respectively. To test the model’s robustness to domain shift, we additionally cycle the remaining 16 cells under the same conditions as above, except now fixing the discharge current for all cells and cycles at 52.5mA (1.5 C) instead of randomly changing the discharge current at each cycle. The space of protocols considered is illustrated in Fig. 2 and an example of the capacity trajectories of three cells is provided in Supplementary Fig. 1 for illustration of the difference from typical monotonic capacity fade experiments. A complete description of cycling protocols is provided in the Methods and the full set of operating conditions that each cell is subjected to is detailed in Supplementary Table 1.

Having used the first dataset to confirm the approach can successfully forecast discharge capacity several cycles ahead, we later significantly expand our analysis to explore the model’s robustness to cell manufacturer, changes to usage pattern and operating temperature. To achieve this, we cycle an additional 48 cells from a second manufacturer, RS Pro (nominal capacity 40 mAh), under a much wider range of usage patterns. In this case, each cell is again subjected to 100 cycles of two-stage CC charging, and one-stage CC discharging, with the three rates randomly selected at the start of each cycle. However, we now make the problem more challenging by having a different distribution of currents for each cell, to replicate the scenario in which different battery users have different average usage patterns to each other, but still exhibit random cycle-to-cycle behaviour. Of these cells, sixteen are also cycled at a higher operating temperature of 35 °C.

### Capacity forecasting using EIS

We first consider the setting in which we want to predict the next cycle discharge capacity, for a cell whose usage history (including for example, cycle or calendar age, or historical capacity–voltage data) is completely unknown, if we apply a particular charging and discharging profile. We frame the problem as a regression task, and train a probabilistic machine learning model to learn the mapping *Q*_*n*_ = *f*(**s**_*n*_, **a**_*n*_), with uncertainty estimates, where **s**_*n*_ is the battery state at the start of the *n*th cycle, **a**_*n*_ is the future action (the *n*th cycle charge/discharge protocol), and *Q*_*n*_ is the discharge capacity measured at the end of the cycle. The battery state vector **s**_*n*_ is formed from the concatenation of the real (${Z}_{{{{{{{{\rm{re}}}}}}}}}$) and imaginary (${Z}_{{{{{{{{\rm{im}}}}}}}}}$) components of the impedance measured at 57 frequencies, *ω*_1_, . . . , *ω*_57_, in the range 0.02Hz-20kHz; ${{{{{{{{\bf{s}}}}}}}}}_{n}=[{Z}_{{{{{{{{\rm{re}}}}}}}}}({\omega }_{1}),{Z}_{{{{{{{{\rm{im}}}}}}}}}({\omega }_{1}),...,{Z}_{{{{{{{{\rm{re}}}}}}}}}({\omega }_{57}),{Z}_{{{{{{{{\rm{im}}}}}}}}}({\omega }_{57})]$. The action vector is formed from the concatenation of the *n*th cycle charge and discharge currents.

**Fig. 3:**
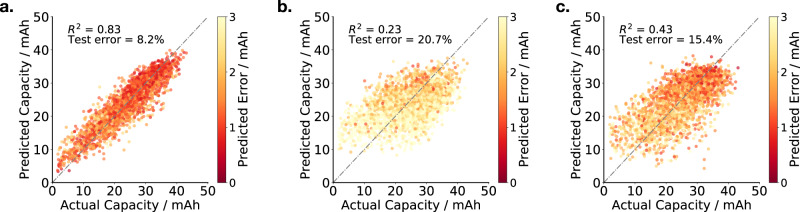
Predicting next cycle discharge capacity. **a** Given knowledge of the state (the battery's internal state, as characterised by the EIS spectrum) and the action (the next cycle charge/discharge protocol), our model predicts the next cycle discharge capacity with an error of 8.2%. Both state and action are needed to accurately forecast performance; using (**b**) state or (**c**) action alone is insufficient.

Figure 3 illustrates the accuracy of our model. Using both state and action as input, the next cycle discharge capacity is predicted with an average error of 8.2%. Importantly, both state and action (Fig. 3a) are found to be necessary to predict future cell performance: if state (Fig. 3b) or action (Fig. 3c) alone are used as inputs, the test error approximately doubles to 20.7% and 15.4% respectively. This demonstrates the importance of both the cell’s internal health and the externally selected usage in determining realised cell performance.

For applications such as optimised charging and repurposing triaging, it is important that a model of battery life trajectory can forecast not only the immediate next cycle discharge capacity, but also capacity several cycles into the future^49,50^. With this in mind, we next investigate how the predictive accuracy of the model changes as we push the model to predict capacity further into the future. In each case, the input comprises the concatenation of the state representation at the start of the *n*th cycle, **s**_*n*_, with the ‘action’ vector **a**_*n*...*n*+*j*_ comprising all charging and discharging currents that will be applied between cycle *n* and cycle *n* + *j*.

**Fig. 4:**
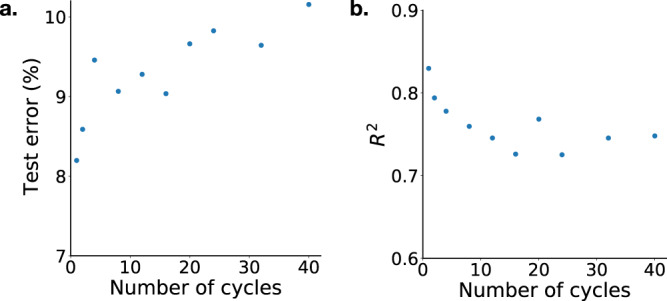
Multi-step forecasting. Our model can also forecast longer term battery performance, as quantified by (**a**) % test error, and (**b**) *R*^2^ value. Given the EIS spectrum and knowledge of the next protocols that will be applied to the cell, the discharge capacity is predicted with a test error of less than 10% up to 32 cycles in advance.

Figure 4 shows how the coefficient of determination *R*^2^ changes with *j*. As expected, the accuracy of the model generally decreases as the forecasting interval increases. However, the model still attains *R*^2^ = 0.75 when projecting 40 cycles into the future.

### Data efficiency and robustness to domain shift

**Fig. 5:**
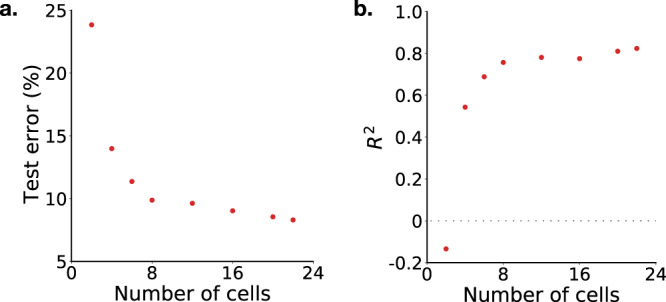
Data efficiency. The model performance, as quantified by (**a**) % test error and (**b**) *R*^2^, improves as the number of cells used to train increases. The model is data-efficient, achieving a test error of less than 10% with just eight cells in the training set.

We next test the robustness of our method by investigating data efficiency and model generalisability. To test data efficiency, we measure how performance changes as the number of cells used to train the model increases. As seen in Fig. 5, there is a marked reduction in test error from 23.8% to 8.2% as the number of cells increases from two to 22. Nevertheless, the model is demonstrably data-efficient, with just eight cells needed to obtain a test error of less than 10%.

**Fig. 6:**
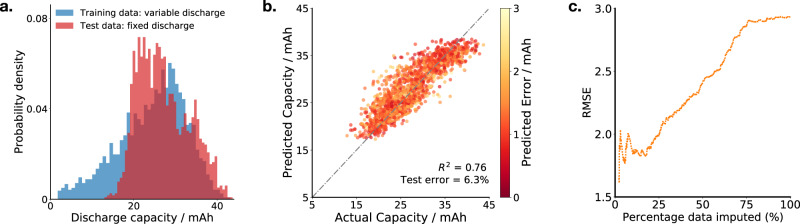
Robustness to domain shift. **a** The distribution of discharge capacity is different for cells cycled under variable discharge rates (blue) compared to a fixed discharge rate (red); the overlap region of the two distributions appears darker in colour. **b** Our model, trained on the variable discharge rate cells, accurately predicts the discharge capacities of cells cycled under a fixed discharge rate. The colour of the plotted points denotes predicted uncertainty (see colour bar). **c** The model `knows what it does not know': when we restrict the test data used to calculate the root-mean squared error (RMSE) by including only the predictions that the model is most confident about (i.e. with lowest predictive uncertainty), the RMSE reduces.

An important test of model generalisability is to study model accuracy when the domain distribution changes, i.e. when the model is being deployed in settings that are different from the training data^12^. This is important for deployment in the field as the approach needs to be robust to driving patterns that might be different from the training data^8^. We test model robustness by cycling an additional 16 cells from the same manufacturer, but now adjusting the cycling protocol by fixing the discharge current to 1.5C for each cell throughout its life. We use a model trained using only cells that were subjected to random discharge currents over their lifetime, to predict next-cycle discharge capacity of cells subjected to fixed discharging. To illustrate the difference in training and test datasets, the distribution of discharge capacities is shown for each in Fig. 6a.

The predictive accuracy of the model on the fixed discharge dataset is illustrated in Fig. 6b. Promisingly, the model attains a test error of just 6.3% on this domain-shifted dataset, which corresponds to *R*^2^ = 0.76.

Our model also outputs predictive uncertainty, which indicates how certain the model is about the quality of its predictions. It is especially important in the domain-shifted setting that the model ‘knows what it does not know’ and estimates high predictive uncertainty about data points that it is likely to obtain a high error on. We can test the model’s ability to estimate its uncertainty by observing how the average test error changes as the number of data points is reduced to include only the data points that the model is most confident about. If a model can successfully estimate its level of certainty, the average test error should reduce as the proportion of data is reduced to include only the most confidently predicted points. Figure 6c shows a 32% reduction in root-mean-squared error (RMSE) as the proportion of data is reduced from 100% to the most confident 25%, demonstrating that our model has learnt which predictions it should be confident about.

### Comparison of state representations

Having demonstrated the ability of the EIS spectrum to capture battery state, we now benchmark this representation of battery health against other approaches utilised in the literature, including the state-of-the-art feature-based method^22,51^, and consider whether there are additional features to the EIS spectrum that can serve to augment battery state. Simple measures that have been used to forecast or estimate battery SOH include using the previous cycle discharge capacity, or the capacity throughput since cycling commenced. More advanced approaches include extracting features of the historical capacity–voltage discharge curves, as shown in Fig. 1. The state-of-the-art approach to extracting such features was implemented by Severson et al.^22^ and inspired the approaches to feature extraction used recently by Attia et al. and Paulson et al.^37,51^. We benchmark how our EIS-based approach performs relative to those state-of-the-art features.

Further, we assess whether incorporation of physical interpretations, in the form of equivalent circuit models (ECM), improves predictions. We use the widely implemented Randles circuit model, comprising a series resistance, connected with a resistance in parallel with a capacitance and a Warburg impedance element, as well as the more complex Extended Randles circuit, which adds an additional resistor-capacitor parallel combination in series to the Randles circuit. The ECM is fitted to the spectrum (at an associated computational cost) and we use the extracted parameters as the state representation instead of raw EIS data.

In total, we consider the following features in our benchmark:

- Previous cycle discharge capacity *Q*_*n*−1_.
- Capacity throughput (CT) since cycling commenced, as defined by the sum of cell charge and discharge capacities from cycles 0 to *n* − 1.
- State of Health (SOH), as defined by *Q*_*n*−1_/*Q*_0_.
- State-of-the-art features of the capacity–voltage discharge curve (CVF): Following Severson et al.^22^, we form a state representation at the start of cycle *n* by extracting features from the capacity–voltage discharge curve after cycle *n* − 1. We fit each curve to a spline function, linearly interpolating to measure capacity at 1000 evenly spaced voltages from ${V}_{\min }$ to ${V}_{\max }$. This 1000-dimensional capacity vector **Q**_*n*−1_ is normalised by subtracting the equivalent vector from cycle 0, **Q**_0_. The following features are then used as inputs: ${V}_{\max }$, ${V}_{\min }$, $\log ({{{{{{{\rm{var}}}}}}}}({{{{{{{{\bf{Q}}}}}}}}}_{n-1}-{{{{{{{{\bf{Q}}}}}}}}}_{0}))$, $\log ({{{{{{{\rm{IQR}}}}}}}}({{{{{{{{\bf{Q}}}}}}}}}_{n-1}-{{{{{{{{\bf{Q}}}}}}}}}_{0}))$. Additionally, we fit the capacity to a sigmoid $Q(\tilde{V})=\frac{{p}_{0}}{1.0+\exp ({p}_{1}(\tilde{V}-{p}_{2}))}$ where $\tilde{V}$ is the normalised voltage and use the parameters *p*_0_, *p*_1_, *p*_2_ as features.
- Equivalent circuit model parameters (ECM-R and ECM-ER): We fit equivalent circuit models using the Randles circuit (ECM-R) and Extended Randles circuit (ECM-ER) to the EIS spectra and concatenate the obtained parameters together.

We note that in contrast to EIS features, the formation of a state representation using the first four aforementioned features demands access to historical current-voltage data, over at least the entirety of the previous discharge and for some features, over the entire cell lifetime. However, they benefit from the advantage of not requiring equipment to measure the EIS spectrum, which comes with an associated financial and temporal cost. Forming a state representation using the ECM parameters (extracted from the EIS spectrum) has an associated computational cost and can be considered a form of dimensionality reduction of the raw EIS data. An additional problem faced by ECMs in general is non-uniqueness, in that multiple different combinations of ECM parameters can generally explain a particular EIS spectrum equally well^52^.

Table 1 shows how the state representation impacts test error and model goodness of fit. In all cases, the model is trained to predict the next cycle discharge capacity, given the next cycle protocol and the chosen state representation. Interrogating the relative importance of features, we first consider the baseline of using EIS only (without including the protocol) and using the protocol only (without including EIS). Perhaps unsurprisingly, battery degradation is a function of both the current state and future charge/discharge protocol. As such, using both EIS and the protocol significantly outperforms using EIS only or using the protocol only.

**Table 1:** Comparison of state representations

Input	*R*^2^	Test error (%)
Protocol only	0.32	15.4
EIS only	0.05	20.7
EIS + Protocol	0.83	8.2
ECM-R + Protocol	0.69	10.7
ECM-ER + Protocol	0.80	8.4
SOH + Protocol	0.61	12.4
CVF + Protocol	0.75	8.8
CT + Protocol	0.52	13.3
*Q*_*n*−1_ + Protocol	0.56	12.1
EIS + CVF + Protocol	0.88	6.7
EIS + CVF + CT + *Q*_*n*−1_ + Protocol	**0.91**	**6.2**

We consider whether augmenting the EIS-based state representation with additional features can enhance model performance. The State of Health (SOH), as defined by the ratio between the previous discharge capacity and the initial discharge capacity, does not fully capture the battery’s current health: using this scalar state representation instead of the multidimensional EIS representation increases the test error by 51%. The most informative representation of battery state is obtained by adding previous capacity–voltage discharge curve features (CVF), capacity throughput (CT), and previous discharge capacity *Q*_n−1_.

We then explore the impact of physics-based representation of the EIS spectrum, using the Randles (ECM-R) and extended Randles (ECM-ER) equivalent circuit models. Comparing EIS + Protocol with ECM-R + Protocol and ECM-ER + Protocol reveals that these physics-based models lose information, and using a machine learning approach to directly learn from raw data might be advantageous.

We next consider the different approaches that have been reported in the literature, *Q*_*n*−1_, SOH, CT, and CVF, with CVF being the state-of-the-art in the battery informatics literature. In all cases, EIS + Protocol outperforms those other features with Protocol, although CVF is competitive.

Interestingly, information from capacity–voltage curve data (CVF) is complementary to EIS - combining EIS with these features leads to a significant increase in accuracy (EIS + CVF + Protocol). This is perhaps unsurprising, as EIS probes the impedance of the single ‘static’ cell discharged state (with high information content per instant state), whilst capacity–voltage curves probe how the cell state evolves continuously over the path from charged to discharged (with low information content per instant state).

Finally, the best model performance is attained by combining all of the above features to form the state representation. In this case the average test error is just 6.2%.

### Robustness to different cell manufacturers

We now extend our analysis to explore how robust our approach is to changing the cell manufacturer, adjusting the operating temperature and adjusting the average use pattern. We repeat our experiment on a new batch of 32 commercial LiR coin cells (of nominal capacity 1 C = 40 mAh) from RS Pro, a different manufacturer, except we now make the problem significantly more challenging by subjecting different subgroups of cells to one of four different usage distributions. These usage distributions are shown in Supplementary Table 1.

We measure the accuracy of the model in two ways: firstly, we consider the case where the model is exposed to cells that have been subjected to the same distribution of protocols as the test set (random splitting), and second, the more challenging case where the model is only trained on the cells which are subjected to three of the cycling protocol distributions and tested on the remaining eight cells subjected to a different cycling protocol. This is a much harder task as the average usage on the test cells is very different to the average usage on the training cells—it is a test of whether the model can extrapolate to different average use not just different cycle-to-cycle use.

The results for different state representations are shown in Table 2 for both the case where the train/test split is random, and where the split is stratified into different usage patterns. Comparable observations are made for cells purchased from the second manufacturer: namely, the most accurate predictions are made when the state representation is formed using features of the EIS spectrum alongside those formed from the discharge curve (CVF). As expected, the model performs significantly better when it has been trained on data from some cells that have been exposed to a similar distribution of cycling patterns as those that the model is tested on. However, the model remains performant in the scaffold split scenario, and in this setting the test error reduces by 30% when the state representation is formed using the EIS spectrum alongside the features of the discharge curve, instead of solely using features of the discharge curve. These additional results further demonstrate that if available, both the EIS spectrum and discharge curve can act as informative markers of the battery’s internal state, but that they are complementary to each other.

**Table 2:** Robustness of approach to cell manufacturer

Input	Random train/test split		Stratified train/test split	
	*R*^2^	Test error (%)	*R*^2^	Test error (%)
EIS + Protocol	0.78	15.2	0.59	21.1
CVF + Protocol	0.69	16.5	0.38	27.7
EIS + CVF + Protocol	0.84	12.9	0.68	19.5

We make qualitatively similar observations when we test our approach on cells manufactured by RS Pro (rather than Powerstream), with EIS found to be a slightly better state representation than state-of-the-art capacity-voltage features (CVF). The best results are obtained when the two representations are combined. We test how the model performs when we split the training and testing sets randomly, and when we instead stratify the training and testing sets such that the model is tested on cells with a different usage distribution to the cells it was trained on. Usage conditions and an extended comparison of different state representations are provided in Supplementary Tables 1, 2 and 3.

We next verify that the model is robust with respect to changing external operating temperature. We cycle an additional 16 cells at 35 °C and test the model trained on data from cells cycled at room temperature. Table 3 shows that our model can extrapolate to cells operated at these higher temperatures, but that the EIS spectrum plays a particularly important role in characterising the battery state when the cell is not operated at the same temperature. The model obtains a test error of 34.2% when only the discharge curve features are used to characterise state, which reduces to 14.0% when both the EIS spectrum and discharge curve features are used. This further demonstrates the additional information that EIS signals contain relative to charging-discharging curves, and supports the hypothesis that EIS implicitly tracks temperature^53^.

**Table 3:** Robustness to operating temperature

Input	*R*^2^	Test error (%)
EIS + Protocol	0.76	14.6
CVF + Protocol	0.20	34.2
EIS + CVF + Protocol	0.80	14.0

Accuracy of the model trained on 32 cells (manufactured by RS Pro) cycled at 23 °C and tested on 16 cells from the same manufacturer but cycled at 35 °C. Here we compare the model accuracy when different representations are used to characterise the battery’s internal state. An extended comparison of different state representations is provided in Supplementary Table 4.

## Discussion

In this paper, we showed that the electrochemical impedance spectrum accurately characterises the internal state of a cell, and a machine learning model can be trained to accurately forecast both immediate and longer term cell performance with predictive uncertainty, even amid uneven and unknown historical cell usage. Our model achieves comparable accuracy (8.2% test error) to the state-of-the-art forecasting approach (8.8% test error) when testing on cells subjected to the same distribution of operating conditions as the cells used to train the model. However, as outlined in Fig. 1, the state-of-the-art approach demands access to historical cycling data whereas our model enables forecasting with no historical data. Additionally, our model significantly outperforms the state-of-the-art model when extrapolating to a higher operating temperature, with a 57% reduction in test error (from 34.2% to 14.6%).

Our method is data-efficient, achieving a next-cycle test error of 9.9% with training data from just eight cells, and is robust to shifts in dataset distributions. Additionally, we find that there is scope to boost model performance by 25% if historical cycling data is available; such data can be used to derive features that augment the cell state representation. We demonstrate that our approach can be utilised across different cell chemistries, and the model is robust to different operating temperatures.

Our approach differentiates from the prior art in two important ways : First, we employ an information-rich electrical signal—EIS—which captures the response of the cell across different timescales without any knowledge of the cycling history. This is in contrast to most existing methods which employ features from the charging–discharging curve—a significantly more coarse-grained signal—as input to machine learning models. Our results suggest significant improvements in battery management systems abound by incorporating circuitries that measure electrochemical impedance, albeit at a financial and temporal cost.

Second, we focus on uneven cycling, where the charging and discharging rates vary from cycle to cycle. This departs from previous studies on machine learning for battery degradation which focused on constant charge/discharge conditions, which are typical in battery testing. Our results problematise the concept of a single scalar State of Health, as the state of the battery is dependent on the extent of the myriad different degradation mechanisms, which in turn depends on the sequence of historic charge/discharge protocols. Rather, we suggest that a cell can be described by a multidimensional state vector, captured using informative high-dimensional measurements like EIS, and a machine learning approach can be used to predict future capacities given the state vector and future charge/discharge protocols. Furthermore, although in this work we only consider forecasting starting from an initially discharged state, we hypothesise that it should be possible in future work to forecast discharge capacity starting from any state of charge based on the EIS measurement, since EIS spectrum implicitly tracks state of charge^54–56^.

We note that the general framework that we have laid out for predicting future battery performance given current cell state and future actions has scope to be applied in a broad range of battery diagnostic and control settings. For example, predicting the effect of a proposed charging protocol on next cycle discharge capacity as well as long term degradation is important for optimising rapid charging applications^51^, where a balance must be achieved between charging time and rate of cell degradation^57^. Our work can additionally be extended to consider more complicated dynamic usage protocols, such as WLTC.

## Methods

### Battery cycling

For this study we cycle 88 commercial LiR coin cells purchased from two different manufacturers, Powerstream and RS Pro, in a temperature regulated laboratory at 23 ± 2 °C. A Biologic BCS-805 potentiostat is used for cycling, and photographs of the experimental setup are provided in Supplementary Fig. 2.

Across all datasets, cells are subjected to a sequence of randomly selected charge and discharge currents for 110–120 full charge/discharge cycles. Cycling commences when the cell is in the fully discharged state, and each cycle comprises the following steps: (a) resting for 20 min at the open circuit voltage, (b) acquisition of the galvanostatic EIS spectrum in the fully discharged state, (c) two stage CC charging, (d) resting for 20 min at the open circuit voltage, (e) acquisition of the galvanostatic EIS spectrum in the fully charged state, (f) one stage CC discharging. The galvanostatic EIS spectrum is always measured by collecting impedance measurements at 57 frequencies uniformly distributed in the log domain in the range 0.02Hz-20kHz using a sinusoidal current with amplitude of 5 mA. Cells are cycled in a temperature-controlled lab room at 23 ± 2 °C.

To generate the first dataset, we cycle 24 Powerstream LiR 2032 coin cells (nominal capacity 1 C = 35 mAh). For these cells, charging consists of a two-stage CC protocol; currents are randomly selected in the ranges 70–140mA (2C–4C) and 35mA-105mA (1C-3C) in stages 1 and 2 respectively. A time limit of 15 min is set for each charging stage such that the total charging time is constrained to be 30 min or less. Charging will stop before the 30 min time limit if the safety threshold voltage of 4.3 V is reached. During discharging, a single constant discharge current, randomly selected in the range 35mA-140mA (1C–4C), is applied, until the voltage drops to 3.0 V.

An additional 16 cells (also manufactured by Powerstream and of nominal capacity 35 mAh) are cycled under the same conditions, except now we fix the discharge current at 52.5 mA (1.5C) for all cells and cycles, instead of randomly changing the discharge current at each cycle.

We then generate a second dataset that enables exploration of the model’s robustness to cell manufacturer, changes to usage pattern and operating temperature. We cycle 48 cells from a second manufacturer, RS Pro (nominal capacity 40 mAh), under a much wider range of usage patterns. The general six-step cycling protocol remains the same as described above, with each cell again being subjected to 100 cycles of two-stage CC charging, and one-stage CC discharging, with the three rates randomly selected at the start of each cycle. However, the distribution of currents now changes for each cell. Of these cells, sixteen are also cycled at a higher operating temperature of 35 ± 2 °C, in a temperature-controlled heating chamber. A description of the full set of operating conditions that each cell is subjected to is detailed in Supplementary Table 1.

### Machine learning model

All problems in this study are framed as regression tasks. We train a probabilistic machine learning model to learn the mapping *Q*_*j*_ = *f*(**s**_*n*_, **a**_*n*...*j*_), with uncertainty estimates, where **s**_*n*_ is the battery state at the start of the *n*th cycle, **a**_*n*_ is the set of future cycling protocols applied over cycles *n* to *j*, and *Q*_*j*_ is the discharge capacity at the end of the *j*th cycle. The battery state vector **s**_*n*_ is generally formed from the concatenation of the real (${Z}_{{{{{{{{\rm{re}}}}}}}}}$) and imaginary (${Z}_{{{{{{{{\rm{im}}}}}}}}}$) components of the galvanostatic EIS spectrum measured in the fully discharged state at the start of the cycle at 57 frequencies, *ω*_1_, . . . , *ω*_57_, in the range 0.02Hz-20kHz; ${{{{{{{{\bf{s}}}}}}}}}_{n}=[{Z}_{{{{{{{{\rm{re}}}}}}}}}({\omega }_{1}),\, {Z}_{{{{{{{{\rm{im}}}}}}}}}({\omega }_{1}),...,\, {Z}_{{{{{{{{\rm{re}}}}}}}}}({\omega }_{57}),\, {Z}_{{{{{{{{\rm{im}}}}}}}}}({\omega }_{57})]$. For the task of predicting next cycle discharge capacity, the action vector **a**_*n*_ is formed from the concatenation of the *n*th cycle charge and discharge currents. When predicting discharge capacity several cycles, *j*, ahead of time, the future protocol is now formed from all charging and discharging currents that will be applied between cycle *n* and cycle *n* + *j*.

For the machine learning model, we use an ensemble of 10 XGBoost models^58^, each with 500 estimators and a maximum depth of 100. The mean and standard deviation of the predictions made by each model in the ensemble are used to quantify the predicted output and the predictive uncertainty. To test model performance we use the median *R*^2^ score and median percentage error. To obtain test metrics from a dataset comprising *N* cells, we randomly leave two test cells out, train on the remaining *N*−2 cells and repeat this process *N*/2 times, leaving different cells out each time.

## Supplementary information

Supplementary Information

## Data Availability

The data generated in this study are provided in the Zenobo database at 10.5281/zenodo.6645536^59^.

## Code availability

The code required to reproduce this manuscript is available at https://github.com/PenelopeJones/battery-forecasting^60^.
