# Supplementary material for: Impedance-based forecasting of lithium-ion battery performance amid uneven usage
Source: Nat Commun. 2022 Aug 16;13:4806. doi: 10.1038/s41467-022-32422-w (PMC9381522; doi:10.1038/s41467-022-32422-w)
Supplement: Supplementary file 1 — Supplementary Information [file 41467_2022_32422_MOESM1_ESM.pdf]

Supplementary Information

Impedance-based forecasting of lithium-ion battery performance amid uneven usage

Penelope K. Jones,[1,2] Ulrich Stimming,[3] and Alpha A. Lee[1]

[1] *Department of Physics, University of Cambridge, Cambridge, UK*

[2] *The Alan Turing Institute, London, UK*

[3] *Chemistry, School of Natural and Environmental Sciences, Newcastle University, Newcastle upon Tyne, UK*

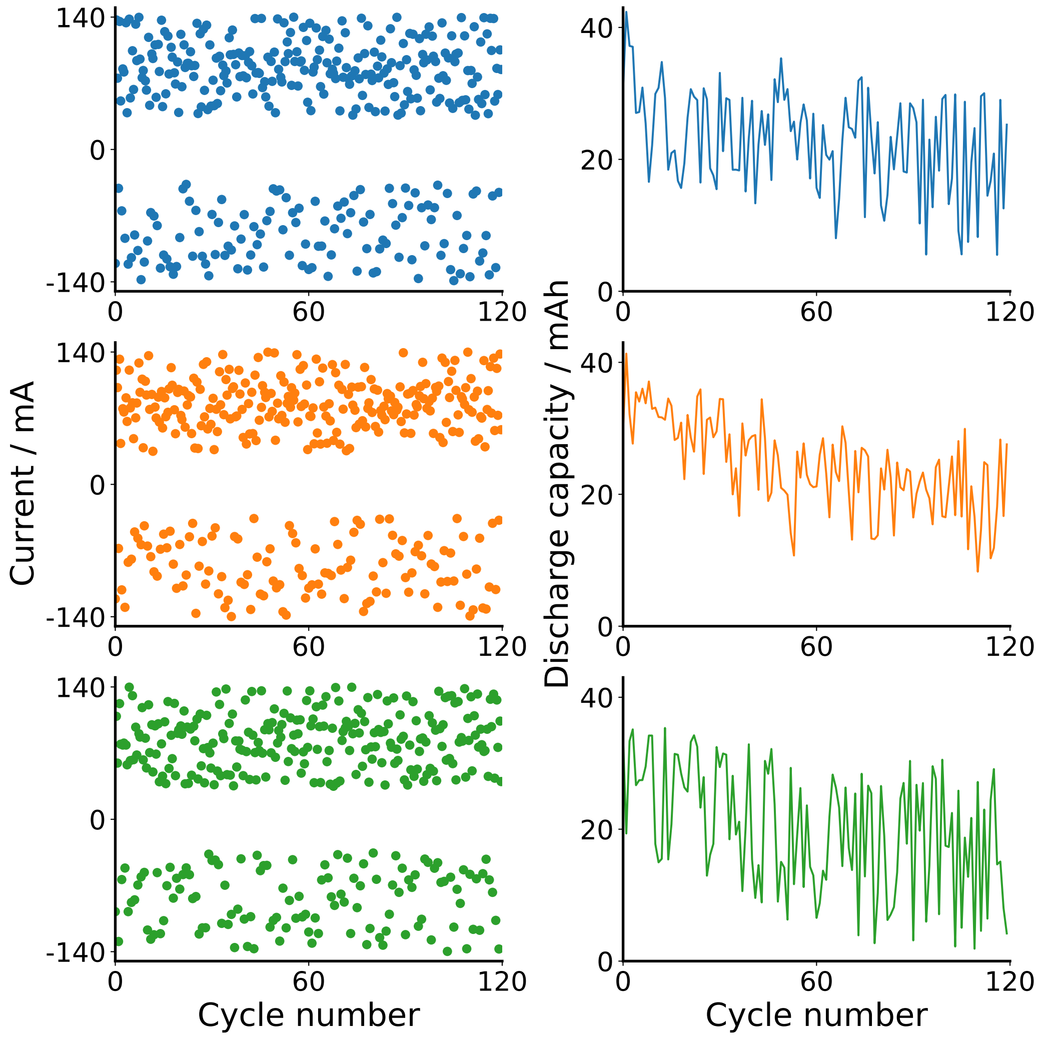

Supplementary Figure 1: **Examples of capacity fade trajectories.** Example of the applied random charge and discharge currents applied to three sample Powerstream cells and the corresponding measured discharge capacity at each cycle. It is seen that the measured discharge capacity does not change in a monotonic way from cycle to cycle because the usage is changing as well as the internal battery state.

a. b.

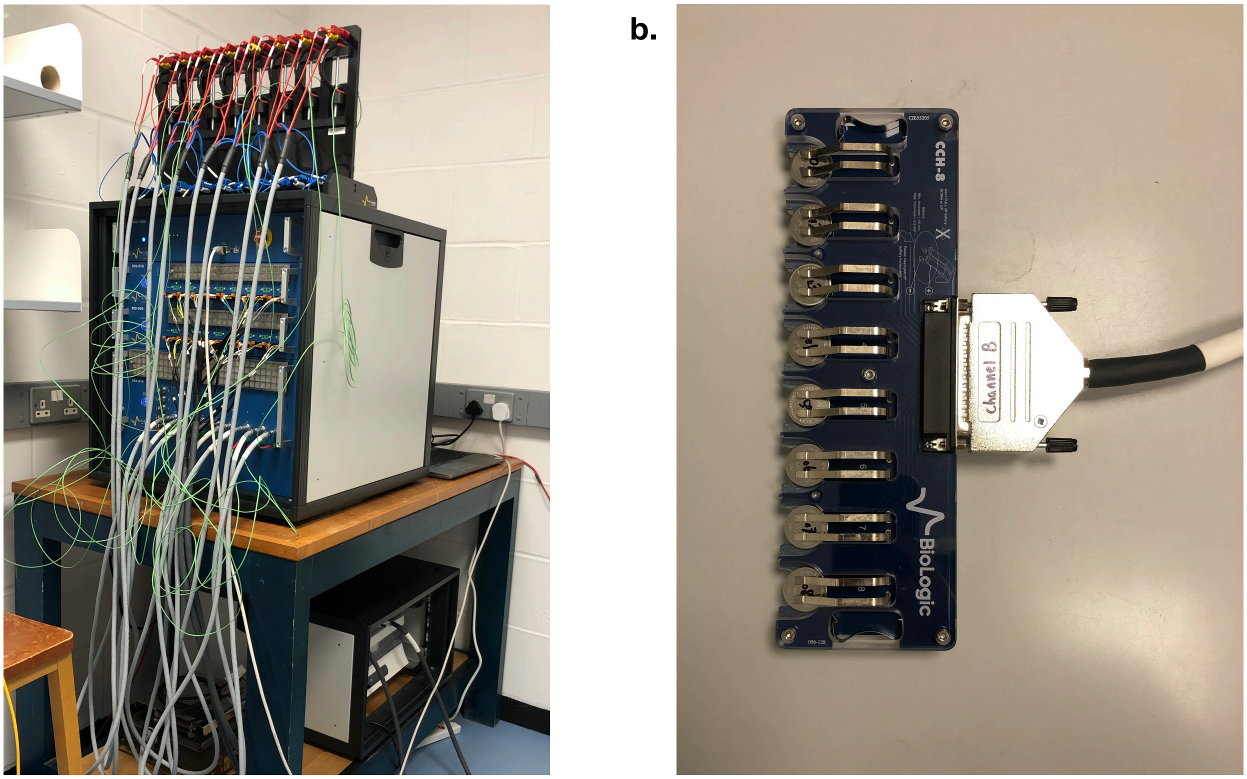

Supplementary Figure 2: **Battery cycling set-up. a.** Coin cells are connected to a Biologic BCS-805 potentiostat. Cells are cycled in a temperature controlled lab room when cycled at 23 °C and in a temperature controlled heating chamber when cycled at 35 °C. **b.** Coin cell holders are provided by Biologic.

Cell IDs	No. of cells	Manufacturer	Stage 1 C-rates	Stage 2 C-rates	Discharge rates	Temperature / °C
PJ097-PJ112; PJ145-PJ152	24	Powerstream	2C-4C	1C-3C	1C-4C	23±2
PJ121-PJ136	16	Powerstream	2C-4C	1C-3C	1.5C	23±2
PJ247-PJ251; PJ264-PJ267	8	RS Pro	2C-4C	1C-3C	1C-4C	23±2
PJ252-PJ255; PJ268-PJ271	8	RS Pro	2C-3C	2C-3C	2C-3C	23±2
PJ256-PJ259; PJ272-PJ275	8	RS Pro	3C-4C	1C-2C	2.5C-4C	23±2
PJ260-PJ263; PJ276-PJ279	8	RS Pro	1.5C-2.5C	1.5C-2.5C	1C-2.5C	23±2
PJ296-PJ311	16	RS Pro	1C-4C	1C-3C	1C-4C	35±1

Supplementary Table 1: **Operating conditions.** Overview of the operating conditions that the cells cycled for this paper are subjected to.

**Input**	$\mathbf{R^2}$	**Test error (%)**
Protocol only	0.43	21.7
EIS + Protocol	0.78	15.2
ECM-R + Protocol	0.68	17.7
ECM-ER + Protocol	0.65	17.9
EIS + CVF + Protocol	0.84	12.9
ECM-R + CVF + Protocol	0.82	13.7
ECM-ER + CVF + Protocol	0.84	14.4
CVF + Protocol	0.69	16.5
Q_{n-1} + Protocol	0.58	18.0
ECM-R + CVF + CT + Q_{n-1} + Protocol	0.83	14.5
ECM-ER + CVF + CT + Q_{n-1} + Protocol	0.87	14.0
EIS + CVF + CT + Q_{n-1} + Protocol	0.84	13.0

Supplementary Table 2: **Robustness of approach to cell manufacturer (random train/test split)**. We test how well our approach generalises to alternative chemistries by cycling 32 additional cells (manufactured by RS Pro). Each cell is subjected to random charging and discharging, according to four possible distributions of protocols (see Supplementary Table 1). Here we assess how the model performs under 16-split validation, where two cells are randomly held out in each split, not necessarily from the same subcategory. We assess how the model performance change when the features used to form the state representation are varied.

**Input**	$\mathbf{R^2}$	**Test error (%)**
Protocol only	0.21	28.9
EIS + Protocol	0.59	21.1
ECM-R + Protocol	0.46	23.7
ECM-ER + Protocol	0.61	23.5
EIS + CVF + Protocol	0.68	19.5
ECM-R + CVF + Protocol	0.70	20.4
ECM-ER + CVF + Protocol	0.67	21.4
CVF + Protocol	0.38	27.7
Q_{n-1} + Protocol	0.20	28.2
ECM-R + CVF + CT + Q_{n-1} + Protocol	0.67	20.5
ECM-ER + CVF + CT + Q_{n-1} + Protocol	0.68	21.5
EIS + CVF + CT + Q_{n-1} + Protocol	0.67	19.9

Supplementary Table 3: **Robustness to cell manufacturer (stratified train/test split)**. We test how well our approach generalises to alternative manufacturers by cycling 32 additional cells (manufactured by RS Pro, nominal capacity 40 mAh). Each cell is subjected to random charging and discharging, according to four possible distributions of protocols (see Supplementary Table 1). Here we assess how the model performs under 4-split validation, with scaffold splitting: for each split 8 cells with one cycling distribution are held out. We assess how the model performance change when the features used to form the state representation are varied.

**Input**	$\mathbf{R^2}$	**Test error (%)**
Protocol only	0.10	36.0
EIS + Protocol	0.76	14.6
ECM-R + Protocol	0.66	17.1
ECM-ER + Protocol	0.69	16.4
EIS + CVF + Protocol	0.80	14.0
ECM-R + CVF + Protocol	0.70	17.1
ECM-ER + CVF + Protocol	0.71	17.1
CVF + Protocol	0.20	34.2
Q_{n-1} + Protocol	0.27	31.8
ECM-R + CVF + CT + Q_{n-1} + Protocol	0.69	17.6
ECM-ER + CVF + CT + Q_{n-1} + Protocol	0.66	18.6
EIS + CVF + CT + Q_{n-1} + Protocol	0.82	13.2

Supplementary Table 4: **Robustness to operating temperature**. Accuracy of the model trained on 32 cells (manufactured by RS Pro) cycled at 23°C and tested on 16 cells (also manufactured by RS Pro) cycled at 35°C. Here we compare the model accuracy when different representations are used to characterise the battery's internal state.
